# Not so weak PICO: leveraging weak supervision for participants, interventions, and outcomes recognition for systematic review automation

**DOI:** 10.1093/jamiaopen/ooac107

**Published:** 2023-01-09

**Authors:** Anjani Dhrangadhariya, Henning Müller

**Affiliations:** Institute of Informatics, University of Applied Sciences Western Switzerland (HES-SO), Sierre, Switzerland; University of Geneva (UNIGE), Geneva, Switzerland; Institute of Informatics, University of Applied Sciences Western Switzerland (HES-SO), Sierre, Switzerland; University of Geneva (UNIGE), Geneva, Switzerland

**Keywords:** weak supervision, machine learning, information extraction, evidence-based medicine

## Abstract

**Objective:**

The aim of this study was to test the feasibility of PICO (participants, interventions, comparators, outcomes) entity extraction using weak supervision and natural language processing.

**Methodology:**

We re-purpose more than 127 medical and nonmedical ontologies and expert-generated rules to obtain multiple noisy labels for PICO entities in the evidence-based medicine (EBM)-PICO corpus. These noisy labels are aggregated using simple majority voting and generative modeling to get consensus labels. The resulting probabilistic labels are used as weak signals to train a weakly supervised (WS) discriminative model and observe performance changes. We explore mistakes in the EBM-PICO that could have led to inaccurate evaluation of previous automation methods.

**Results:**

In total, 4081 randomized clinical trials were weakly labeled to train the WS models and compared against full supervision. The models were separately trained for PICO entities and evaluated on the EBM-PICO test set. A WS approach combining ontologies and expert-generated rules outperformed full supervision for the participant entity by 1.71% macro-F1. Error analysis on the EBM-PICO subset revealed 18–23% erroneous token classifications.

**Discussion:**

Automatic PICO entity extraction accelerates the writing of clinical systematic reviews that commonly use PICO information to filter health evidence. However, PICO extends to more entities—PICOS (S—study type and design), PICOC (C—context), and PICOT (T—timeframe) for which labelled datasets are unavailable. In such cases, the ability to use weak supervision overcomes the expensive annotation bottleneck.

**Conclusions:**

We show the feasibility of WS PICO entity extraction using freely available ontologies and heuristics without manually annotated data. Weak supervision has encouraging performance compared to full supervision but requires careful design to outperform it.

Lay Summary

Systematic reviews are clinical summaries, incredibly resource-consuming to produce and involve redundant document filtering processes that machine learning could automate. Manual PICO (participant, intervention, comparator, outcome) information analysis aids document filtering but is one of the most resource-intensive stages for writing systematic reviews. Supervised machine learning based PICO information extraction could accelerate document filtering but requires massive hand-labelled datasets for training. We propose a weak supervision approach that uses more than 127 freely available vocabularies and expert-designed rules to label 4081 documents in evidence-based medicine (EBM)-PICO dataset with PICO information. Powerful pretrained transformer models were fine-tuned for PICO extraction using these programmatically labelled documents and compared to the results using expensive hand-labelled EBM-PICO documents. The token-level macro-F1 score was used to compare full supervision and weak supervision. We also examined the errors in the EBM-PICO training data and rectified them in the EBM-PICO test set. The weak supervision approach had promising results overall and had a better F1 score than full supervision for the participant information, albeit it required a careful design. We further rectify the errors made by weak supervision and intend to improve the methodology. Finally, adopting weak supervision for highly compositional PICO information is challenging but feasible and extensible to more clinical entities.

## INTRODUCTION

Systematic reviews (SR) are an evidence-based practice of answering clinical questions using a transparent and quantitative approach. The reviewers must collect as many candidate publications as possible, identify the relevant publications, and integrate their results via statistical meta-analysis. A clinical SR question is typically formulated using the PICO (participants, interventions, comparators, outcomes) framework, for example, “Will aerobic exercise (Intervention) improve fatigue (Outcome) in cancer patients (Participant) compared to usual care (Comparator)?.” A publication is only relevant for answering a question if it studies the selected participants, interventions (and their comparators), and outcomes.^1^ Manually analyzing PICO information from thousands of publications for a single SR often takes 2–8 months of 2 medical experts’ time.^2^ It can be automated using machine learning (ML) by directly pointing the human reviewers to the PICO descriptions, facilitating quick decision-making for the study’s relevance.

Supervised ML requires hand-labeled data, but hand-labelling data with PICO information require people with combined medical and informatics skills, which is expensive and time-consuming in terms of intensive annotator training and the actual annotation process. Labelling PICO information is tricky because of the high disagreement between human annotators on the exact spans constituting PICO, leading to human errors in hand-labeled corpora.^3^ Some studies examine the errors in the publicly available evidence-based medicine (EBM)-PICO benchmark.^4–6^ More importantly, depending upon the SR question, PICO criteria are extended to PICOS (S—study design), PICOC (C—context), PIBOSO (B—background, O—other), and PICOT (T—timeframe).^1^^,^^7^^,^^8^ Hand-labeled datasets are static and prohibit quick manual re-labelling in case of human errors or when a downstream task requires new entities. This annotation bottleneck has pivoted attention toward weakly supervised (WS) learning that relies on programmatic labelling sources to obtain training data. Programmatic labelling is quick and allows efficient modifications to the training data labels per the downstream application changes.

WS learning has demonstrated strengths for clinical document classification and relation extraction, but clinical entity extraction tasks have heavily relied on fully supervised (FS) approaches.^9–14^ Despite the availability of Unified Medical Language System (UMLS), a large compendium of medical ontologies, which can be re-purposed for weak entity labelling, it has not been extensively applied to clinical entity labelling.^15^ Several legacy clinical applications are also supported by rule-based if-else systems relying on keyword cues that aid weak labelling.^16–18^ With so many weak labelling sources available, the challenge for weak supervision is efficiently aggregating these sources of varying accuracy. Compare this to crowdsourcing, where an important task is to model the worker's accuracy without the ground truth.^19^ Though crowdsourcing requires annotator training and quality control, programmatic labelling does not.^20^

Data programming is a domain-agnostic generative modeling approach combining multiple weak labelling sources and estimating their accuracies. The effectiveness of data programming for biomedical entity recognition has been explored by Fries et al^21^ in their Trove system. However, Trove only explores well-defined entities like chemical, disease, disorder, and drug. PICO categories are highly compositional spans by definition, fuzzier in comparison and much more intricate in that they can be divided into subclasses. A shortcoming of span extraction is that even after a machine points a human reviewer to the correct PICO span, the reviewer requires to manually read and understand its finer aspects to screen the study for relevance. Span extraction hence leads to semiautomation but hinders full-automation. The entity recognition approach to PICO is not as easy as the entity recognition approach to disease or chemical names, which are more or less standardized. PICO terms are not standard, and even the experts disagree on the exact tokens constituting them.^3^ WS PICO entity recognition has not garnered as much attention as supervised span recognition. As far as our knowledge goes, only 2 studies exist for WS PICO recognition. One of these approaches only explores distant supervision for intervention extraction using a single labelling source.^22^ The other approach studies weak supervision for PICO span extraction but still utilizes some supervised annotation signals about whether a sentence includes PICO information.^23^

The challenges to developing weak supervision approaches to PICO entity recognition are first defining the subclasses within PICO spans and then mapping several available ontologies and terminologies to these. The next challenge is developing WS classifiers by optimally combining ontologies and evaluating their performance compared to full supervision. Another challenge is developing higher-cost expert-generated rules corresponding to these subclasses to aid ontology classifiers and evaluate their combined performance. We also identified limitations in the currently available EBM-PICO training dataset and corrected them in the EBM-PICO test set for reliable evaluation of the WS approaches. Our work demonstrates the feasibility of using weak supervision for PICO entity extraction using the EBM-PICO benchmark and shows how weak supervision overtakes full supervision in certain instances.

## METHODOLOGY

**Figure 1.**
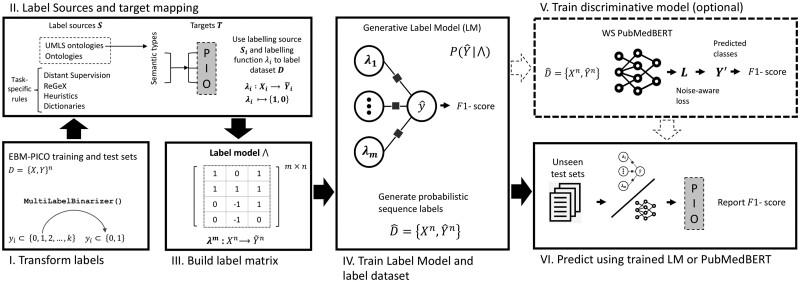
Weak PICO entity extraction approach. (I.) Multi-class labels in the EBM-PICO benchmark are binarized. (II.) Low-cost UMLS vocabularies are re-purposed as labelling sources and experts design rules as high-cost labelling sources. (III.) Labelling functions map the training sequences to class labels using labelling sources resulting in an m×n label matrix. (IV. and V.) The label matrix is used to train a generative model that outputs probabilistic labels that a downstream transformer model can use for entity recognition.

The birds-eye view of our approach is shown in Figure 1.

### Datasets

EBM-PICO is a widely used dataset with multi-level PICO annotations: span-level or coarse-grained and entity-level or fine-grained (refer to Table 1). Span-level annotations encompass the maximum information about each class. Entity-level annotations cover the more fine-grained information at the entity level, with PICO classes further divided into semantic subclasses. The dataset comes predivided into a training set (*n* = 4933) annotated through crowd-sourcing and an expert annotated gold test set (*n* = 191) for evaluation.^4^ The EBM-PICO annotation guidelines caution about variable annotation quality (https://www.ncbi.nlm.nih.gov/pmc/articles/PMC6174533/bin/NIHMS988059-supplement-Appendix.pdf). Abaho et al^5^ developed a framework to *post hoc* correct EBM-PICO outcomes annotation inconsistencies. Lee and Sun^6^ studied annotation span disagreements suggesting variability across the annotators. Low annotation quality in the training dataset is excusable, but the errors in the test set can lead to faulty evaluation of the downstream ML methods. We evaluate ∼1% of the EBM-PICO training set tokens to gauge the possible reasons for the fine-grained labelling errors and use this exercise to conduct an error-focused PICO re-annotation for the EBM-PICO gold test set. The dataset is pretokenized and did not require additional preprocessing except the addition of POS tags and token lemma using spaCy (https://spacy.io/). Multi-class fine-grained PICO annotations were binarized, that is, a token label was reset to 1 if the token represented a fine-grained entity.

**Table 1.** P (participant), I (intervention), and O (outcome) represent the coarse-grained labels that are further divided into respective fine-grained labels

	P	I/C	O
0	No label	No label	No label
1	Age	Surgical	Physical
2	Sex	Physical	Pain
3	Sample size	Drug	Mortality
4	Condition	Educational	Side effect
5		Psychological	Mental
6		Other	Other
7		Control	

*Note*: The table is taken from Nye et al^4^

### Binary token labelling

Automatic PICO entity labelling is a classical binary token labelling problem whereby a function maps an input sequence of *n* text tokens, $X=(x_{1},x_{2},\ldots ,\;x_{n})$ to output sequence $Y=(y_{1},y_{2},\ldots ,\;y_{n})$, where $y_{i}\subset y;y=\{1,0\}$ is the label for token xi. In weak supervision, Y is latent and should be estimated by aggregating several weak labelers of variable accuracy. The estimates $\hat{Y}$ of Y are assigned as probabilistic token labels of X leading to a weakly labelled dataset that can be used to train discriminative models.

### Labelling functions

In a binary token labelling task, a labelling function (LF) is a weak classifier λ that uses domain-specific labelling sources S and a logic to emit token labels $\tilde{Y_{i}}$ with labels $\tilde{y_{i}}\in \{-1,0,+1\}$ for a subset of input $X_{i}$ tokens. An LF designed for a particular target class t ∈ T (here; T⊂ {participant, intervention, outcome}) should output 1 for the positive token label, 0 for the negative token label, and abstain (−1) on the tokens where it is uncertain λ ↦{-1,0,+1}. We designed 3 LF types depending on the types of labelling sources. (1) The ontology or dictionary LFs for a target class take a dictionary of terminologies mapped to one of *y* ⊂{0,+1} token labels. Any LF using ontologies or dictionaries used string matching as the labelling heuristic. Relevant bigram word co-occurrences were used to account for fuzzy span matching from the terminologies. A bigram was considered relevant for a vocabulary if it occurred ≤25 times in that vocabulary. (2) A regular expression (ReGeX) LF for a target class takes regex patterns for {0,+1} labels and abstains from the rest. (3) A heuristic LF is personalized for each target class and takes a generic regex pattern and specific part-of-speech (POS) tag signals. Abbreviations in clinical studies are considered using a heuristics abbreviation identifier, and the identified abbreviations were mapped to their respective target classes. Stopwords from Natural Language Toolkit (NLTK) (https://www.nltk.org), spaCy, Gensim (https://radimrehurek.com/gensim/), and scikit learn (https://scikit-learn.org) were used to initialize negative token label templates.

### Labelling sources

This section describes the labelling sources S used and their mapping to the PICO targets T. We used the 2021AB-full release of the UMLS Metathesaurus English subset with 223 vocabularies. After removing non-English and zoonotic vocabularies and the vocabularies containing fewer than 500 terms, we remained with 127 vocabularies.^15^ Terms in the selected vocabularies were preprocessed by removing stopwords, numbers, and punctuation. Additional vocabularies included disease ontology; human phenotype ontology; ontology of adverse events; chemical entities of biological interest; comparative toxicogenomics database chemical and disease subclasses; clinical trials ontology; gender, sex, and sexual orientation ontology; chemotherapy toxicities ontology; cancer care: treatment outcomes ontology; symptoms ontology; nonpharmacological interventions ontology; and nursing care coordination ontology.^24–34^ ReGeX and heuristics like POS tag cues were used to capture recurring class-specific patterns otherwise not captured by standardized terminologies. Vocabularies are structured, standardized data sources that do not capture writing variations from clinical literature and custom-built ReGeX are restricted by either task or entity type.^35^^,^^36^ We used distant supervision dictionaries created from the structured fields of clinicaltrials.gov (CTO) as described by Dhrangadhariya and Müller^22^ Principal investigators of the clinical study manually enter data in CTO, thereby incorporating large-scale writing variations.^37^

### Sources to targets

Along with the source S and the logic to map $S_{i}$ to token labels, an LF needs information about which target $T_{i}$ label and binary token class to map the source. We report how the LF sources were mapped to PICO targets in this section. UMLS 2021AB-full release contains 16 543 671 concept names, making direct concept to PICO target mapping impractical. These concepts are organized under semantic type categories (eg, disease, sign and symptoms, and age group) (https://www.nlm.nih.gov/research/umls/META3_current_semantic_types.html), which allows mapping these semantic categories to PICO targets, invariably mapping the concepts from the vocabularies to target classes.^37^ It is a challenging expert-led activity, though decomposing PICO into subclasses greatly helps map sources to target. A semantic category was marked 1 to represent a positive token label for that target class or 0 to represent a negative token label for that target class. Non-UMLS vocabularies were obtained from NCBO bioportal (https://bioportal.bioontology.org/) and were chosen to be PICO target specific and assigned to a single label. Target-specific distant supervision dictionaries were created from the structured fields of clinicaltrials.gov (CTO). The structured field “condition or disease” was mapped to the participant target, and the “intervention/treatment” field was mapped to the intervention target. The semistructured “primary outcome measures” and “secondary outcome measures” fields were mapped to the outcome target. The hand-coded dictionaries were designed using the official websites listing patient-reported outcome (PROMs) questionnaires (https://www.thoracic.org/members/assemblies/assemblies/bshsr/patient-outcome/) and PROMs (https://www.safetyandquality.gov.au/our-work/indicators-measurement-and-reporting/patient-reported-outcomes/proms-lists). Other hand-crafted dictionaries were separately designed for participant gender and sexuality, intervention comparator terms.

### LF aggregation

Depending upon the number of sources S for each T, we had several LFs. Each LF $\lambda_{i}\in \;\Lambda_{m};\;\Lambda =\{\lambda_{1},\;\lambda_{2},\;\ldots ,\;\lambda_{m}\}\;$maps a subset of inputs $X^{n}$ to output sequence ${\tilde{Y}}^{n}$ with labels $\tilde{y}\in \left\{-1,0,+1\right\}$ yielding a label matrix $\lambda \subset {\left\{-1,0,+1\right\}}^{m\times n}$. The weakly generated labels might have conflicts and overlaps and are generally noisy. The LFs can be ensembled using the majority vote (MV) rule, where a token label is elected only when a majority of $\lambda_{i}$ vote for it. Ties and abstains lead to the selection of the majority label.

(1) $${\hat{Y}}_{\mathrm{MV}}=\underset{y\;\subset \;\left\{0,1\right\}}{\max}\sum_{i=1}^{m}1\left(\lambda_{i}=\;y_{i}\right).$$

However, MV considers each LF as conditionally independent and does not consider the variable accuracies of different labelling sources weighing them equally. Snorkel implements data programming paradigm into the label model (LM) that re-weights and aggregates LFs into probabilistic labels ${\hat{y}}_{i}$. To do this, the LM trains a generative model $P\left(\Lambda ,Y\right)$ to estimate LF accuracies $\theta_{j}$ using stochastic gradient descent to minimize log loss in the absence of labelled data.^35^^,^^38^ Although the ground truth is not observable to estimate accuracies; they can be estimated using observed agreement and disagreement rates between LF pairs $\lambda_{i}$, $\lambda_{j}\;$in Λ. Generative modeling ultimately results into token label probabilities $\hat{Y}$ for label classes $\left\{0,1\right\}$. GridSearch was used to fine-tune the parameters of the LM using the hand-labeled validation set from the EBM-PICO. The parameters are listed in the Experimental details section of the Supplementary Material. Once we have the pseudo-labels generated by majority voting or the LM, these could be used to train a discriminative model.

(2) $$\hat{\theta }=\underset{\theta }{\mathrm{argmin}}\left(-\log\sum_{Y}p_{\theta }\left(\Lambda ,\;Y\right)\right).$$

### Experiments

The LFs $\lambda_{m}$ were used to label the EBM-PICO training set and obtain λ. We tested MV and LM to aggregate LFs. LM output probabilistic labels for the training set were used as weak supervision signals to train downstream PubMedBERT to minimize noise-aware cross-entropy loss. PubMedBERT was trained on PubMed literature and was chosen because of its domain similarity to our training data (PubMed abstracts) and task.^39^ It was tuned on fixed parameters listed in the experimental details section in the Supplementary Material.

(3) $$\hat{\omega }=\underset{\omega }{\mathrm{argmin}}\frac{1}{N}\sum_{i=1}^{n}E_{\hat{y}∼\hat{Y}}\left[l\left(f\left(x,\omega \right),\;\hat{y}\right)\right]$$

UMLS ontologies are readily available sources of weak supervision, while searching the non-UMLS ontologies requires an additional effort and understanding of the target class and domain. On the contrary, designing the rules requires understanding the idiosyncratic clinical patterns for the target classes. Therefore, we experiment and report results on 3 “expense” tiers to gauge the performance changes: (1) UMLS labelling sources, (2) UMLS and non-UMLS labelling sources, and (3) UMLS, non-UMLS and expert-generated rules. We test label aggregation via MV and LM along with WS PubMedBERT for the above tiers. The WS experiments were compared against a competitive FS PubMedBERT trained using the hand-labeled EBM-PICO training set. For all the experiments, 80% of the EBM-PICO dataset was used for training and 20% for validation.

UMLS ontologies were ranked based on the total number of *n*-gram overlaps between the respective terminology and the EBM-PICO validation set. These were then partitioned into 127 partitions $s=\left(1,\;2,\;\ldots ,\;127\right)$, where the first partition combined the entire UMLS into a single LF and was used as the baseline. The last partition kept all the terminologies as separate LFs.

Partition-wise performance over the validation set was tracked.

### Evaluation

We report the classical macro-averaged F1 and recall for MV, LM, and WS transformer models and the FS PubMedBERT models. Token-level macro-F1 was chosen to make it comparable to the PICO extraction literature. Mean macro-averaged scores are reported over 3 runs of each model, with the top 3 random seeds (0, 1, and 42) used in Python. The models were separately trained for each target class recognition task using the raw (inside, out) tagging scheme. We used Student’s *t*-test with an alpha α threshold of 0.05 to measure the statistical significance.

## RESULTS

### PICO decomposition

**Figure 2.**
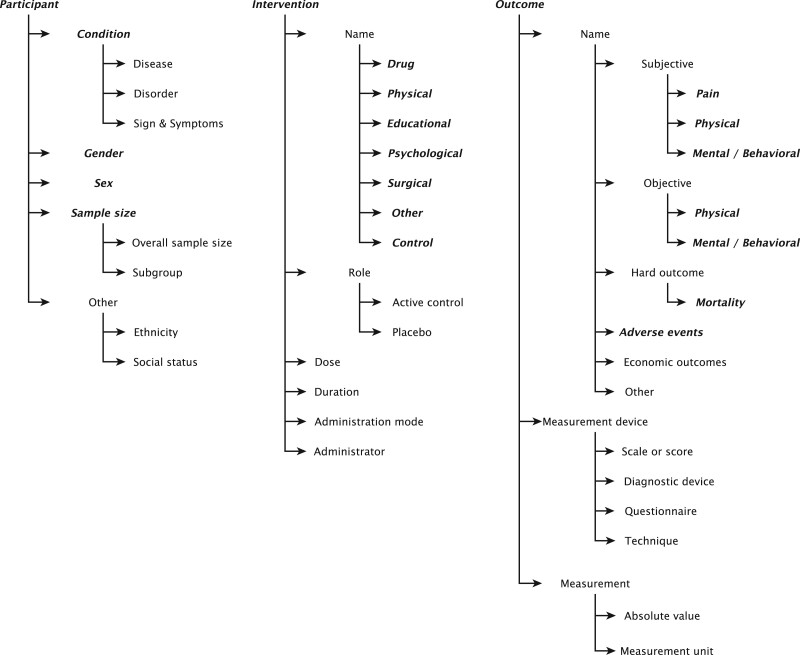
Hierarchical representation of PICO subclasses. The categories marked in bold–italic are the same as the fine-grained categories in the EBM-PICO corpus.

We extended the EBM-PICO subclasses (refer to Table 1) to better query the labelling sources and design LFs (see Figure 2). For a more comprehensive subgrouping, we propose developing a PICO ontology.^40^ It is more straightforward to search for ontologies representing adverse events or diseases rather than fending for an ontology describing the entire participant or outcome spans. It is easier to grasp cues separately for outcome terms and instruments of outcome measurement to develop heuristics. The intervention span can include the intervention name, role (primary intervention or comparator), dosage, frequency, mode of administration, and administrator. The outcome span can include the outcome names, the scales, techniques or instruments used to measure them, and the absolute outcome measurement values. The EBM-natural language processing guidelines restrict annotating the outcome name, how it was measured, and the intervention’s name and role (control, placebo), leaving out the other subclasses.

### Error rectification

**Figure 3.**
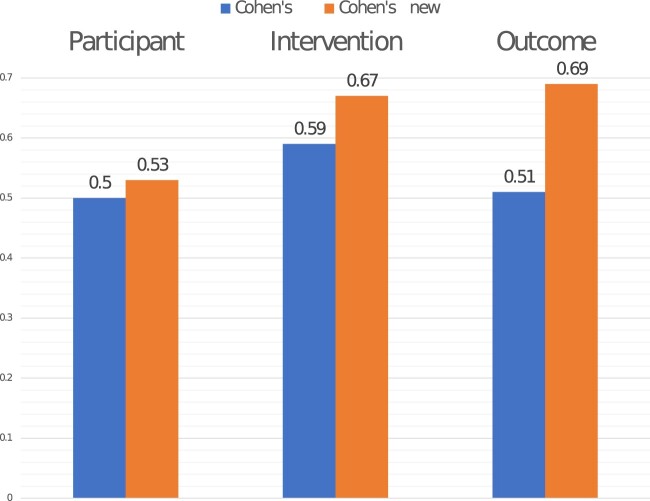
Cohen's $\kappa_{\mathrm{new}}$ between the expert annotated EBM-PICO gold test set and EBM-PICO compared to the Cohen's κ for EBM-PICO gold test set annotations.

We rectified the errors in the EBM-PICO validation set and categorized them for each PIO class, as shown in Table 2. Of 12 960 (∼1% of 1 303 169) training tokens evaluated to gauge the errors, 18.30% of the intervention class tokens, 23.39% of the participant class tokens, and 20.21% of the outcome class tokens were errors. These error categories are elaborated in the Supplementary Material. The error analysis was used to correct fine-grained annotation errors in the EBM-PICO test set, and both the EBM-PICO and its updated version were used for evaluation. We were constrained with obtaining multiple annotators for the re-annotation to calculate inter-annotator agreement. Therefore, we calculated Cohen's $\kappa_{\mathrm{new}}$ between the original EBM-PICO gold set and our re-annotation over 200 documents and compared it to Cohen's κ (see Figure 3) provided by the authors of the original corpus.^4^

**Table 2.** Error distribution and error categories in the analyzed tokens (∼1%) of the EBM-PICO corpus

Error category	Participant	Intervention	Outcome
Repeat mention unmarked	213	227	207
Remain un-annotated	47	59	71
Inconsistency	46	18	85
Punctuation/article	15	23	48
Conjunction connector	30	36	57
Junk	53	79	30
Extra information	80	146	58
Generic mention	70	120	85
Total errors	554	708	641

Table 3 reports macro-averaged F1 for the experiments detailed in the experiments section compared to the FS approach. Error rectification leads to an overall average F1 improvement of 4.88% across the experiments using a weakly labeled training set with the highest average improvement of 8.25% (7.15–9.52%) for participants and 2.68% (−0.11% to 4.28%) for outcomes. For the participant class, both the LM and the WS F1 scores increase the full supervision score by 0.90–1.71%. It has to be noticed that weak supervision outperforms full supervision on the rectified benchmark only for the participant entity.

**Table 3.** Macro-averaged F1 scores for UMLS, UMLS + other, and rule-based weak supervision

Target	LF source	#LF	MV		LM		WS		FS	
			Fine	Corr	Fine	Corr	Fine	Corr	Fine	Corr
P	UMLS	3–4	62.13	69.28	64.28	72.22	65.32	73.49	72.99	74.41
	+Ontology	4	61.72	69.32	64.23	72.18	64.76	72.31		
	+Rules	19–119	63.08	72.06	65.79	75.31	66.73	**76.12**		
I/C	UMLS	8–95	59.7	63.94	60.11	64.28	59.17	61.72	**83.37**	81.06
	+Ontology	5–101	62.14	66.92	62.83	67.09	67.06	69.76		
	+Ru1es	4–35	58.51	63.45	64.34	68.17	70.27	72.39		
O	UMLS	5–6	55.79	59.85	58.76	62.36	60.83	63.55	**81.2**	80.53
	+Ontology	4–5	56.006	59.64	59.27	62.34	59.55	60.46		
	+Rules	3–5	55.08	59.36	60.9	62.87	60.5	60.39		

*Note*: Underlined values show the best score without manually labelled training data. Bold values show the best overall F1 score in any category. Fine: EBM-PICO fine-grained annotations; Corr: EBM-PICO fine-grained annotations (EBM-PICO updated). I/C: intervention/comparator; O: outcome; P: participant.

#### MV versus LM versus WS

**Figure 4.**
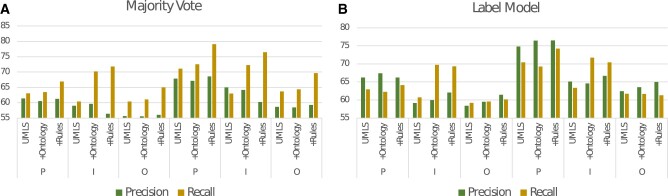
Precision and recall across the experiments for the (A) majority vote models (left) and (B) label models (right).

The LM improved the average performance by 2.74% (0.17–5.83%) compared to majority voting. However, PubMedBERT did not guarantee improved performance across the targets leading to performance drops between 0.4 and 2.56%. Though the WS PubMedBERT models did not always improve the performance compared to their LM counterparts, they had the best F1 score for each target class. The majority voting had higher recall across experiments compared to precision, while LM focused on precision (see Figure 4), making it a possible choice for recall-oriented PICO extraction tasks.

#### LF tiers

Adding non-UMLS LFs to the UMLS tier increases performance for the intervention target by an average of 4.48% but leads to performance drops for the participants and outcomes targets by 0.36% and 0.64%, respectively. Adding task-specific LFs increased the overall F1 by a negligible 0.98%. Heuristics improved performance for the interventions LM by 11.1%.

#### UMLS partitions

**Figure 5.**
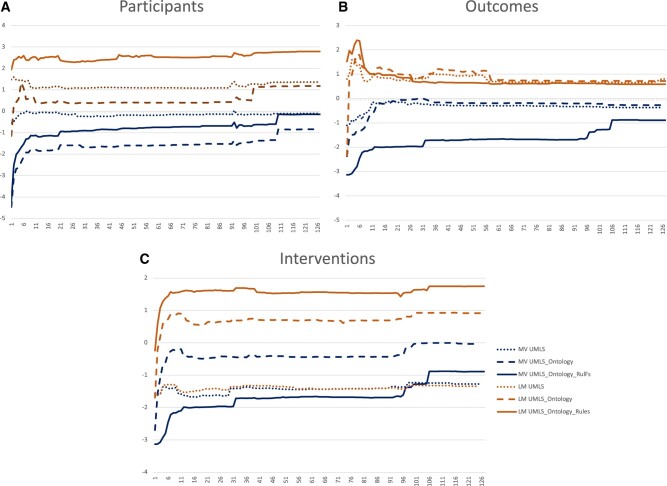
The relationship between the number of UMLS partitions and the macro-averaged F1 score for (A) participants target, (B) outcomes target, and (C) interventions target.

To investigate the optimal number of UMLS LFs required, we used the same methodology as in Trove, holding all non-UMLS and heuristics LFs fixed across all ablation tiers and computed performance across $s=\left(1,\;2,\;\ldots ,\;127\right)\;$partitions of the UMLS terminology. We noticed an increased performance for the first few partitions. However, we did not see the performance drop with a further increase in the participant and intervention target partition number. Partitions with more than 100 LFs performed better. This situation contrasts with Trove, where an increase in partitions leads to a drop in performance across targets (see Figure 5). For the outcomes target, an increase in the number of partitions leads to an increased performance initially but a drop with a further increase in the partition numbers. LM outperforms MV on training performance across the 2 targets and experiments except for the intervention target, where the MV model combining UMLS and additional ontologies outperforms LM. The simple baseline collapsing UMLS into a single LF usually did not perform better than the others in UMLS partitions for any of the 3 experiment tiers (refer to the #LF columns in Table 3).

## DISCUSSION

Our study results show the promising performance of weak supervision compared to full supervision, surpassing it for participant extraction. It has to be noticed that weak supervision requires careful LF design consideration to surpass full supervision, primarily due to the compositional nature of PICO classes. In another study, we use this weak supervision approach to successfully extend PICO to PICOS extraction (S—study type) without needing additional annotated “study type” data to quickly power applications.^41^

Although it is easy to re-purpose the vocabularies for labelling, it is challenging to map them to the correct PICO targets. A decreased or stagnant F1 after adding non-UMLS LFs to the UMLS tiers indicates this. Task-specific ReGeX and heuristics were developed upon inspection of the most frequent terms in the EBM-PICO validation set. The same procedure was followed across the targets. However, the performance boost using rule-based LFs was only observed in the participants and interventions and was detrimental to the outcomes.

Even though LM improves performance compared to MV, MV has a higher recall across experiments indicating a good corpus coverage of the LFs (refer to Figure 4). While some studies press on PICO extraction being a recall-oriented task, this is debated in practice. In practice, high recall might lead to an accompanying high false-positive (FP) rate, leading to the reviewers spending more time to manually weed out FP noise than reading and annotating the abstract with the entities.^23^

LM only considers the information encoded in the weak sources to label phrases from the training text but does not consider the contextual information around the phrases. Transformers consider the contextual information and should generalize beyond the LMs in theory. It is empirically confirmed by the performance boost that PubMedBERT brings this on top of the LM for some instances, but the WS outcomes extraction results refute it.

### Error analysis

We conducted an error analysis on 18 (*n* = 5291 tokens) out of 200 EBM-PICO gold test set documents to contextualize the weak supervision models. Table 4 shows token-level errors divided into either of the 4 classes: (1) false negative (FN)—if the entire entity that the token was part of was missed out by the LM, (2) FP—if the entire entity that the token was part of was falsely recognized as an entity, (3) boundary error (BE)—if the boundary tokens were missed out, but otherwise the entity was identified, and (4) overlapping error—if LM made an error in the nonperipheral tokens of an otherwise identified entity mention. Nonperipheral tokens are all tokens except the first and last of the multi-token entity.

**Table 4.** Distribution of the token-level errors made by the best label models on EBM-PICO gold

	FP	FN	BE	OE
Participants	160	76	80	10
Interventions	308	119	60	0
Outcomes	233	306	139	7

In future, we aim to reduce FNs and dig deeper into this category. Besides participant disease, tokens representing participant sample size, age group, gender, and symptoms subclasses went unrecognized. The LM labeled these FNs with low confidence, meaning the LFs did encode this information, but the signals were not strong enough for correct classification. Such FNs could be mitigated by weighting LFs for these subclasses. Considerable standard and unusual abbreviation terms were missed out, especially the ones encompassed by brackets, for example, metabolic syndrome (MetS), testicular cancer + testicular self-examination, and left ventricular hypertrophy (LVH). The model did not pick some of the standard abbreviations, for example, LVH and MetS, due to a faulty mapping of these abbreviations to the incorrect PICO target.

A similar pattern was observed for the intervention (eg, inference-based approach and radiotherapy) and outcomes class abbreviations too. The mismapping is now amended. LM did not capture the abbreviations enclosed in a bracket (eg, “(COPD)”) as the LFs were not designed to tag these brackets.

Intervention LMs did not recognize common drug names, for example, Fenofibrate and CP-529414.

In addition, many nonstandardized treatment names went unrecognized, for example, substance abuse prevention program, inference-based approach, high-concentration contrast agents, and epigastric impedance. Such terms are absent from UMLS and non-UMLS vocabularies leading to FNs, so the LFs do not encode them. Similarly, intervention BEs were the nonidiosyncratic tokens partially misrecognized because the vocabulary did not encode this partial information.

For example, the term “internal stenting” is partially recognized because “stenting” is a UMLS concept but not “internal.” Similarly, in the term “endopyelotomy stent placement,” only the UMLS concept token “stent” was identified. Participant BE FNs were usually the extra information that described more about the participant's disease, for example, the information about disease staging went unrecognized in the participant's disease entity (in “advanced carcinoma,” the word “advanced” was a BE FN). Such entities not encoded by the LFs contribute to the FNs and could be mitigated by adding relevant vocabulary and rules.^42^ It is straightforward to add vocabulary but challenging to map a semantic group or a vocabulary to PICO categories, especially for the outcomes class. Our current source-to-target mapping approach is manual and based on subjective expert judgment, which an objective algorithm can improve. This mapping could have led to several unexplainable errors, especially for the outcomes and, to a less extent, for the intervention class. In addition, it took time to identify semantic categories and UMLS vocabularies corresponding to the study outcomes pointing toward the gap in developing one.

## CONCLUSION

We adapted weak supervision for PICO spans and developed models for predicting PICO entities without a hand-labeled corpus. We also identified errors pertinent to the current PICO benchmark, updated the dataset, and used both datasets to evaluate the recognition models. The approach achieves promising performance compared to full supervision and warrants further research into weak supervision for compositional entities like PICO. The approach can be extended to more clinical SR entities without a manually labeled corpus, thereby being a starting point to overcome the annotation bottleneck. In the future, we will work on extending the data programming approach to inspect strategies for objectively mapping ontologies to PICO subclasses and experiment using external models like MetaMap as LFs.

## Supplementary Material

ooac107_Supplementary_Data

## Data Availability

The code is available on GitHub (https://github.com/anjani-dhrangadhariya/distant-PICO) and will be made public upon publication. The EBM-PICO error analysis file for the validation set, the error-rectified EBM-PICO gold test set, and other resources like distant supervision CTO dictionaries and hand-crafted dictionaries are available on DRYAD (https://doi.org/10.5061/dryad.ncjsxkszr). More information can be found in the supplementary file.
