# Supplementary material for: Not so weak PICO: leveraging weak supervision for participants, interventions, and outcomes recognition for systematic review automation
Source: JAMIA Open. 2023 Jan 9;6(1):ooac107. doi: 10.1093/jamiaopen/ooac107 (PMC9828146; doi:10.1093/jamiaopen/ooac107)
Supplement: ooac107_Supplementary_Data [file ooac107_supplementary_data.docx]

**Not So Weak-PICO: Leveraging weak supervision for Participants, Interventions, and Outcomes recognition for systematic review automation**

**Anjani Dhrangadhariya^1,2^, Henning Müller^1,2^**

^1^Informatics Institute, University of Applied Sciences Western Switzerland (HES-SO), Sierre, Switzerland

^2^University of Geneva (UNIGE), Geneva, Switzerland

*Corresponding author: Anjani Dhrangadhariya, Rue de Technopôle 3, Informatics Institute, University of Applied Sciences Western Switzerland (HES-SO), 3960 Sierre, Switzerland; anjani.dhrangadhariya@hevs.ch; +41 58 606 90 03

**﻿UMLS SOURCES TO PICO TARGETS**

The UMLS concepts are organized under semantic type categories, an internal semantic ontology provided by UMLS. These semantic categories or types were mapped to the PICO targets as defined in Table 1, along with the justification for the mapping.

| Semantic Type | P | I | O | Reason for choosing |
| --- | --- | --- | --- | --- |
| Mental or Behavioral Dysfunction | 1 | 0 | 1 | Concepts under this semantic group can be participant (condition) dysfunction or an outcome endpoint (clinical manifestation) that could be measured. |
| Sign or Symptom | 1 | 0 | 1 | Concepts under this semantic group could be the improvements in participant symptoms being measured as an outcome. |
| Age Group | 1 | 0 | 0 | Concepts under this semantic group are relevant for participant age but are clearly not intervention or outcome concepts. |
| Disease or Syndrome | 1 | 0 | 0 | Concepts under this semantic group are relevant for participant condition but are clearly not intervention or outcome concepts. |
| Injury or Poisoning | 1 | 0 | 0 | Concepts under this semantic group are relevant for participant condition but are clearly not intervention or outcome concepts. |
| Neoplastic Process | 1 | 0 | 0 | Concepts under this semantic group are relevant for participant condition but are clearly not intervention or outcome concepts. |
| Patient or Disabled Group | 1 | 0 | 0 | Concepts under this semantic group are relevant for participant condition but are clearly not intervention or outcome concepts. |
| Population Group | 1 | 0 | 0 | Concepts under this semantic group can include writing variations for participant gender, sex, social status and other characteristics. |
| Acquired Abnormality | 1 | 0 | 0 | Concepts under this semantic group are relevant for participant condition but are clearly not intervention or outcome concepts. |
| Anatomical Abnormality | 1 | 0 | 0 | Concepts under this semantic group are relevant for participant condition but are clearly not intervention or outcome concepts. |
| Congenital Abnormality | 1 | 0 | 0 | Concepts under this semantic group are relevant for participant condition but are clearly not intervention or outcome concepts. |
| Occupational Activity | 1 | 0 | 0 | Concepts under this semantic group could describe other relevant characteristics of the RCT participants. |
| Professional or Occupational Group | 1 | 0 | 0 | Concepts under this semantic group could describe other relevant characteristics of the RCT participants. |
| Geographic Area | 1 | 0 | 0 | Concepts in this semantic group can be related to ethnicity of a participant or the geographic location from where participants were enrolled |
| Language | 1 | 0 | 0 | Concepts in this semantic group can be related to participants native language. |
| Group | 1 | 0 | 0 | This semantic group is a parent node to all the other groups added under the participant class. |
| Family Group | 1 | 0 | 0 | Could be RCT participant characteristics as well. I abstain on it because I am not sure what other kind of concepts could be encompass |
| Group Attribute | 1 | 0 | 0 | Concepts in this semantic group can describe RCT participant groups like pregnant women, smokers, addicts, *etc*. |
| Pathologic Function | 1 | 0 | 0 | Concepts under this semantic group are relevant for participant condition but are clearly not intervention or outcome concepts. |
| Body Location or Region | -1 | -1 | -1 |  |
| Body Part, Organ, or Organ Component | -1 | -1 | -1 |  |
| Body Space or Junction | -1 | -1 | -1 |  |
| Cell | -1 | -1 | -1 |  |
| Cell Component | -1 | -1 | -1 |  |
| Tissue | -1 | -1 | -1 |  |
| Embryonic Structure | -1 | -1 | -1 |  |
| Body System | -1 | -1 | -1 |  |
| Fully Formed Anatomical Structure | -1 | -1 | -1 |  |
| Anatomical Structure | -1 | -1 | -1 |  |
| Receptor | -1 | -1 | -1 |  |
| Animal | -1 | -1 | -1 |  |
| Human | -1 | -1 | -1 |  |
| Mammal | -1 | -1 | -1 |  |
| Vertebrate | -1 | -1 | -1 |  |
| Plant | -1 | -1 | -1 |  |
| Research Activity | -1 | -1 | -1 |  |
| Carbohydrate Sequence | -1 | -1 | -1 |  |
| Classification | -1 | -1 | -1 |  |
| Conceptual Entity | -1 | -1 | -1 |  |
| Entity | -1 | -1 | -1 |  |
| Environmental Effect of Humans | -1 | -1 | -1 |  |
| Event | -1 | -1 | -1 |  |
| Functional Concept | -1 | -1 | -1 |  |
| Human-caused Phenomenon or Process | -1 | -1 | -1 |  |
| Idea or Concept | -1 | -1 | -1 |  |
| Machine Activity | -1 | -1 | -1 |  |
| Manufactured Object | -1 | -1 | -1 |  |
| Molecular Function | -1 | -1 | -1 |  |
| Molecular Sequence | -1 | -1 | -1 |  |
| Natural Phenomenon or Process | -1 | -1 | -1 |  |
| Nucleotide Sequence | -1 | -1 | -1 |  |
| Organism | -1 | -1 | -1 |  |
| Organization | -1 | -1 | -1 |  |
| Phenomenon or Process | -1 | -1 | -1 |  |
| Physical Object | -1 | -1 | -1 |  |
| Spatial Concept | -1 | -1 | -1 |  |
| Cell or Molecular Dysfunction | -1 | 0 | -1 |  |
| Occupation or Discipline | -1 | 0 | -1 |  |
| Organism Attribute | -1 | 0 | -1 |  |
| Mental Process | -1 | 0 | -1 |  |
| Organ or Tissue Function | -1 | 0 | -1 |  |
| Physiologic Function | -1 | 0 | -1 |  |
| Body Substance | -1 | 0 | -1 |  |
| Organism Function | -1 | 0 | -1 |  |
| Social Behavior | -1 | 0 | -1 |  |
| Biologic Function | -1 | 0 | -1 |  |
| Health Care Related Organization | -1 | -1 | 0 |  |
| Inorganic Chemical | -1 | -1 | 0 |  |
| Fungus | -1 | 0 | 0 |  |
| Virus | -1 | 0 | 0 |  |
| Bacterium | -1 | 0 | 0 |  |
| Medical Device | 0 | 1 | 1 | The concepts under this semantic group can include medical devices used for diagnostic tests or could be the medical devices used to carry out an intervention. Adding this semantic group could increase recall. |
| Enzyme | 0 | 1 | 1 | Concepts under this semantic group could be a part of enzyme therapy interventions. Enzymes and related proteins are essential biological molecules which could serve as disease biomarkers (outcomes). |
| Hormone | 0 | 1 | 1 | Concepts under this semantic group could be a part of hormone therapy interventions. Hormones could also serve as disease biomarkers (outcomes). |
| Finding | 0 | 0 | 1 | Concepts under this semantic group could be the subjective or objective outcome measures of a participant. For example, Smoking cessation behaviour (CUI:C2586-181). |
| Laboratory or Test Result | 0 | 0 | 1 | Concepts under this semantic group could be the subjective or objective outcome measures of a participant. |
| Clinical Attribute | 0 | 0 | 1 | An observable or measurable clinical attribute can be a patient outcome. |
| Qualitative Concept | 0 | 0 | 1 | Concept under this semantic group could include names of measurable or qualitative outcome concepts. |
| Intellectual Product | 0 | 0 | 1 | Concepts under this semantic group could include the official names of outcome measurement scales or questionnaires which also constitute a part of this entity. |
| Quantitative Concept | 0 | 0 | 1 | Concepts under this semantic group could include measurable outcome concepts. |
| Behavior | 0 | 0 | 1 | Behaviour concepts could be measurable or immeasurable RCT outcomes. For example, Treatment Compliance (CUI:C4319828). |
| Individual Behavior | 0 | 0 | 1 | Individual behaviour concepts could be measurable or immeasurable RCT outcomes. For example, Treatment Compliance (CUI:C4319828). |
| Laboratory Procedure | 0 | -1 | -1 |  |
| Gene or Genome | 0 | 0 | -1 |  |
| Research Device | 0 | 0 | -1 |  |
| Indicator, Reagent, or Diagnostic Aid | 0 | 0 | -1 |  |
| Cell Function | 0 | 0 | -1 |  |
| Biomedical or Dental Material | 0 | 1 | 0 | Concepts under this semantic group are a part of intervention class. |
| Clinical Drug | 0 | 1 | 0 | Concepts under this semantic group are a part of intervention class. |
| Pharmacologic Substance | 0 | 1 | 0 | Concepts under this semantic group are a part of intervention class. |
| Therapeutic or Preventive Procedure | 0 | 1 | 0 | Concepts under this semantic group are a part of intervention class. |
| Vitamin | 0 | 1 | 0 | Concepts under this semantic group could form a part of diet and supplement interventions. |
| Health Care Activity | 0 | 1 | 0 | Concepts under this semantic group are related to activities relating to the care of the patients which can be used either as control intervention or even an intervention in RCTs. |
| Daily or Recreational Activity | 0 | 1 | 0 | Concepts under this semantic group could form a part of physical or behavioral intervention or can be used as placebo controls. |
| Educational Activity | 0 | 1 | 0 | Concepts under this semantic group could form a part of educational or behavioral interventions in the RCTs. |
| Immunologic Factor | 0 | 1 | 0 | Concepts under this semantic group could include vaccine names. |
| Diagnostic Procedure | 0 | 1 | 0 | Concepts under this semantic group could be interventions or diagnosis instruments used in diagnostic RCTs |
| Antibiotic | 0 | 1 | 0 | Concepts under this semantic group are a part of intervention class. |
| Chemical Viewed Functionally | 0 | 1 | 0 | Concepts under this semantic group could be active compounds or IMPs of intervention used in RCT. |
| Biologically Active Substance | 0 | -1 | 0 |  |
| Chemical Viewed Structurally | 0 | -1 | 0 |  |
| Food | 0 | -1 | 0 |  |
| Temporal Concept | 0 | -1 | 0 |  |
| Self-help or Relief Organization | 0 | -1 | 0 |  |
| Activity | 0 | -1 | 0 |  |
| Substance | 0 | -1 | 0 |  |
| Chemical | 0 | -1 | 0 |  |
| Organic Chemical | 0 | -1 | 0 |  |
| Nucleic Acid, Nucleoside, or Nucleotide | 0 | -1 | 0 |  |
| Amino Acid, Peptide, or Protein | 0 | -1 | 0 |  |
| Fish | 0 | 0 | 0 |  |
| Amphibian | 0 | 0 | 0 |  |
| Reptile | 0 | 0 | 0 |  |
| Regulation or Law | 0 | 0 | 0 |  |
| Bird | 0 | 0 | 0 |  |
| Eukaryote | 0 | 0 | 0 |  |
| Hazardous or Poisonous Substance | 0 | 0 | 0 |  |
| Element, Ion, or Isotope | 0 | 0 | 0 |  |
| Amino Acid Sequence | 0 | 0 | 0 |  |
| Biomedical Occupation or Discipline | 0 | 0 | 0 |  |
| Professional Society | 0 | 0 | 0 |  |
| Archaeon | 0 | 0 | 0 |  |
| Governmental or Regulatory Activity | 0 | 0 | 0 |  |
| Genetic Function | 0 | 0 | 0 |  |
| Experimental Model of Disease | 0 | 0 | 0 |  |
| Drug Delivery Device | 0 | 0 | 0 |  |
| Molecular Biology Research Technique | 0 | 0 | 0 |  |

**Table 1:** Table details map the semantic categories listed under the semantic type column to the PICO targets. Note: +1 means the concepts under this type will map matching text spans to a positive token label for that target class. 0 represents the concepts under this semantic type will map the matching text spans to the negative token label for that target class. -1 stands for the abstains.

**NON UMLS SOURCES**

In this section, we provide Table 2 with links to the following non-UMLS labelling sources used in our work: Disease Ontology (DO), Human Phenotype Ontology (HPO), Ontology of Adverse Events (OAE), Clinical Trials Ontology (NDD-CTO), Chemical entities of biological interest (ChEBI), Comparative Toxicogenomics Database (CTD) Chemical and Disease subclasses, Gender, Sex, and Sexual Orientation Ontology (GSSO), Chemotherapy Toxicities Ontology (ONTOTOX), Cancer Care: Treatment Outcomes Ontology (CCTOO), Symptoms Ontology (SYMP), Non-pharmacological Interventions Ontology (NPI), Nursing Care Coordination Ontology (NCCO).

| Target class | Ontology | Source |
| --- | --- | --- |
| P | DO | https://bioportal.bioontology.org/ontologies/DOID |
| P | HPO | https://bioportal.bioontology.org/ontologies/HP |
| O | OAE | https://bioportal.bioontology.org/ontologies/OAE |
| I | ChEBI - Chemical | https://bioportal.bioontology.org/ontologies/CHEBI |
| P | ChEBI - Disease | https://bioportal.bioontology.org/ontologies/CHEBI |
| O | CTD | http://ctdbase.org/} |
| P | GSSO | https://bioportal.bioontology.org/ontologies/GSSO |
| O | CCTOO | https://bioportal.bioontology.org/ontologies/CCTOO |
| O | ONTOTOX | https://bioportal.bioontology.org/ontologies/ONTOTOX |
| O | NDD-CTO | https://bioportal.bioontology.org/ontologies/CTO |
| P | SYMP | https://bioportal.bioontology.org/ontologies/SYMP |
| I | NPI | https://bioportal.bioontology.org/ontologies/NPI |
| I | NCCO | https://bioportal.bioontology.org/ontologies/NCCO |

**Table 2.** The table lists the links for the non-UMLS ontologies used in work along with the PICO (P = Participant, I = Intervention and O = Outcome) target class the ontology was mapped.

**﻿LF statistics**

In Table 3, we show the number of LFs for each target class based on the target designated labelling sources.

| LF source | Participant | Intervention | Outcome |
| --- | --- | --- | --- |
| UMLS | 127 | 127 | 127 |
| non-UMLS | 9 | 8 | 8 |
| Distant supervision (CTO) | 1 | 2 | 1 |
| ReGeX/Heuristics | 22 | 3 | 6 |
| Hand-crafted dictionaries | 1 | 1 | 1 |

**Table 3.** The table enumerates the number of labelling functions for each PICO target class labelling sources.

**EXPERIMENTAL DETAILS**

All the code was written in Python v3.8. The label model was initialized with a cardinality of two for positive and negative labels for each target class. Snorkel v0.9.7 was used to train the label model. GridSearch was used to tune the parameters. The parameters used to tune the Snorkel label model are listed in Table 4. All ﻿the NER experiments in this article were conducted in PyTorch v1.9.0, and the models were trained for 15 epochs with a mini-batch size of 10 for training and 6 for evaluation. The maximum sequence length was set to 510 because of the transformer’s limit. For weakly and fully supervised experiments, the EBM-PICO training set was divided, 80% of the data was used for training and 20% for development. ﻿The [CLS] embeddings ﻿from the PubMedBERT layer were used as features of the input text. PubMedBERT was fine-tuned by not freezing weights during the experiments. ReLU was used as the activation function before feeding [CLS] outputs to the linear layer. The gradients were clipped to 1.0 to mitigate the problem of exploding gradients. Each experiment was carried out on a single Quadro RTX 6000 GPU without data and model parallelization. Further parameters used to train the weakly supervised PubMedBERT model are listed in Table 3.

| Parameters | Values |
| --- | --- |
| Learning rate | 0.001, 0.0001 |
| L2 regularization | 0.001, 0.0001 |
| Epochs | 50, 100, 200, 600, 700, 1000, 2000 |
| Precision | 0.6, 0.7, 0.8, 0.9 |
| Optimizer | adamax, adam, sgd |
| Learning rate scheduler | constant |

﻿

**Table 4.** Parameters used to tune the Snorkel label model using GridSearch

| Parameters | Values |
| --- | --- |
| Learning rate | 0.0005 |
| Learning rate warmup. | 0.1 |
| Epsilon | 0.00000001 |
| Epochs | 15 |
| Max sequence length | 510 |
| Optimizer | AdamW |

﻿

**Table 5.** Parameters used to train the PubMedBERT-linear model

**ERROR RECTIFICATION AND ANALYSIS**

We expound on the error categories identified from the EBM-PICO error reactivation exercise and provide examples here. Some of our error categories are as identified by Abaho et al., but we add more classes on top of them. While they limit their error exploration to the outcomes class, we extend it to the rest. An error falls under repeated mention if one instance of an entity is marked, but another identical instance of the same entity in the same context is not marked within the abstract. The reason can be the EBM-PICO guidelines flaw where the annotators of fine-grained entity annotation were confined to only annotate within the longer span-level annotation. Hence, any annotation error missed by the coarse-grained annotators was continued by the fine-grained annotators.

An error falls under *remains unannotated* if a token should have been annotated as an entity but was not. In the intervention class, a large portion of this category was constituted by the generic mentions of controls (placebo, saline), which were not annotated. In the participant class, patient ethnicity and other information like smoking status and pregnancy (marked in the coarse-grained span) were not marked in the fine-grained entity. The reason could be that there was no fine-grained class to categorize this information. The annotators missed multiple important outcomes and repeated mentions in the outcome class.

*Conjunction connector* errors are the conjunctions occurring between two semantically separate entities but are falsely marked as entities. For example, “Nausea and vomiting” are two different outcomes marked as one by annotating the conjunction between them. When falsely marked as an entity, punctuation succeeding the entity, an article, or a preposition preceding the entity fall under the *punctuation/article errors*. Extraneous tokens marked along with the entity tokens fall under the *extra information* category. For example, in the phrase “This trial demonstrated short-term efficacy of smokeless tobacco in combination with”, the annotators had marked “short-term efficacy of smokeless tobacco” as an outcome entity, but only “short-term efficacy” is an outcome entity. In contrast, “smokeless tobacco” is an intervention entity. In the intervention class, the annotation guidelines mentioned not annotating any part of the text that did not mention the intervention name. The annotators often marked extraneous information like intervention dosage, frequency, route of administration and information about the intervention administrator.

A *generic reference* is a co-reference of an entity mentioned using different or similar (but not identical) words. A generic reference of an entity (and its repeated mention) in the same abstract was several times left unmarked by the annotators constituting a generic reference error. For example, if the outcome endpoint “smoking cessation” was referred to in the same abstract elsewhere as “quitting smoking”, it was not marked even though it is a reference to the outcome phrase “smoking cessation”. If “aerobic exercise” mentioned as “exercise intervention” was not marked, this also constitutes a generic reference error. For instance, in an RCT, if the “breast cancer risk counselling” intervention was referred to as “risk counselling”, the former was marked, and the latter was missed. This error was pronounced specially for the non-pharmaceutical interventions and outcomes.

An *inconsistency error* arises when an entity is fully marked in some abstract’s vs when in the other abstracts, the same entity is partially marked. For example, if an exercise intervention involved aerobic exercise involving stretching and running, this information (“stretching”, “running”) was marked in some studies while not in all the other studies. In the case of the participant class, the sample size sub-grouping information was inconsistently marked. In another example, participant sample size information was either partially or completely marked (“59” vs “59 subjects”, “200” vs “200 controls”). We consider a phrase as a sample size only when the absolute value quantifying sample size follows an appropriate unit (subject, controls, patients, participants, women, men). Only in some cases, when the unit was unavailable, did we consider a standalone number as a sample size participant span. In the outcome class, the annotation guidelines marked “what was measured and how it was measured”. Often, the method used for measuring outcomes was inconsistently marked.

A *junk error* is a token entirely irrelevant to an entity but is marked as one. For example, in the outcome’s evaluation, the phrase “evaluate and compare” from the larger phrase “This study aimed to evaluate and compare” was marked as an outcome entity even though it is not a valid outcome.

These errors and inconsistencies in the EBM-PICO gold test (and training) set can cause faulty evaluation of the machine learning approaches defying the purpose of the corpus. A possible reason behind these inconsistencies in the corpus could be that the annotators had clinical background but lacked an informatics background. This situation could undermine the importance of semantic consistency required for annotating such corpora. Hiring annotators with the combined knowledge of clinical and informatics domains might improve the manual annotation quality. Aggregation of crowd annotations for spans with fuzzy span boundaries might lead to many boundary errors, for example, when two disparate entities are linked by a conjunction connector.
